# Contralateral Axillary Lymph Node Metastases Detected by Repeat Sentinel Lymph Node Biopsy in a Patient with Ipsilateral Breast Tumor Recurrence: A Case Report

**DOI:** 10.70352/scrj.cr.26-0507

**Published:** 2026-10-22

**Authors:** Naotaka Uchida, Takako Suda

**Affiliations:** Department of Breast and Endocrine Surgery, Matsue City Hospital, Matsue, Shimane, Japan

**Keywords:** breast cancer, contralateral sentinel lymph node, ipsilateral breast, recurrence, repeat sentinel lymph node biopsy

## Abstract

**INTRODUCTION:**

There is no consensus on the significance of repeat sentinel lymph node (SLN) biopsy in a patient with ipsilateral breast tumor recurrence (IBTR).

**CASE PRESENTATION:**

A 49-year-old premenopausal woman developed an ipsilateral breast cancer (BC) lesion after previously undergoing lumpectomy, SLN biopsy, and adjuvant radiation therapy for left-sided lobular carcinoma *in situ* at the age of 35. Because of the contralateral SLNs identified on radioisotope lymphoscintigraphy, we decided to perform a left mastectomy with contralateral axillary lymph node dissection. Intraoperative SLN mapping using indigo carmine was negative. A total of 3 contralateral axillary lymph node metastases, all sentinel nodes, were identified among 18 resected nodes. Because this patient’s second BC was an invasive ductal carcinoma, a different histology from her first tumor, we diagnosed a new primary BC along with contralateral axillary lymph node metastases and planned adjuvant therapy accordingly.

**CONCLUSIONS:**

Repeat SLN biopsy for IBTR plays a key role in determining accurate SLN status and in optimizing adjuvant therapy.

## Abbreviations

ALND
: axillary lymph node dissection
ASCO
: American Society of Clinical Oncology
BC
: breast cancer
BCS
: breast-conserving surgery
BRCA
: breast cancer susceptibility gene
ER
: estrogen receptor
ESMO
: European Society for Medical Oncology
FDG-PET–CT
: fluorodeoxyglucose PET–CT
HER2
: human epidermal growth factor receptor 2
IBTR
: ipsilateral breast tumor recurrence
IDC
: invasive ductal carcinoma
LCIS
: lobular carcinoma *in situ*
NCCN
: National Comprehensive Cancer Network
PR
: progesterone receptor
RT
: radiation therapy
SLN
: sentinel lymph node

## INTRODUCTION

SLN biopsy is an established procedure for patients with clinically node-negative early BC. When SLNs are negative for metastasis, standard protocols advise omission of ALND.^1)^ Because SLNs are usually ipsilateral to the BC lesion, contralateral axillary lymph node metastases are typically recognized as distant metastases, not regional lymph nodes, based on general rules for clinical and pathologic BC staging issued by the Japanese Breast Cancer Society.^2)^

When IBTR occurs after BCS, repeat SLN biopsy may be indicated. However, previous ALND and RT are associated with a lower detection rate for SLNs and a higher rate of extra-axillary lymphatic drainage pathways. One study showed that lymphatic drainage pattern rates to the contralateral axilla are 25% in patients who have undergone ipsilateral ALND vs. 18.2% in those who have not.^3)^ Similarly, 64.3% of patients with a history of RT demonstrated contralateral axillary drainage vs. 5.3% in the non-RT group.^3)^ The 2022 Japanese Breast Cancer Society clinical practice guidelines for BC state that repeat SLN biopsy may be considered for patients who have not undergone ALND but is not recommended after ALND.^2)^ However, the clinical utility of repeat SLN biopsy remains unclear.

Herein, we describe our experience with a patient who had positive contralateral axillary SLNs detected by preoperative lymphoscintigraphy for a second, new primary BC. When the metastases to the contralateral axilla were confirmed as regional lymph node metastases, we were able to plan for appropriate adjuvant therapy.

## CASE PRESENTATION

A 49-year-old premenopausal woman was referred to our hospital after mammography detected a spiculated mass in her left breast. Her past medical history included left central BC with a histologic diagnosis of LCIS at the age of 35 (**Fig. 1A**). At that time, she underwent left lumpectomy as BCS with SLN biopsy and subsequent RT. No lymph node metastases were identified in the 2 SLNs removed during the surgery. The resection margin was negative. Immunohistochemical staining showed tumor cells that were positive for ER and PR receptors and negative for HER2 overexpression. She received follow-up for 10 years.

**Fig. 1:**
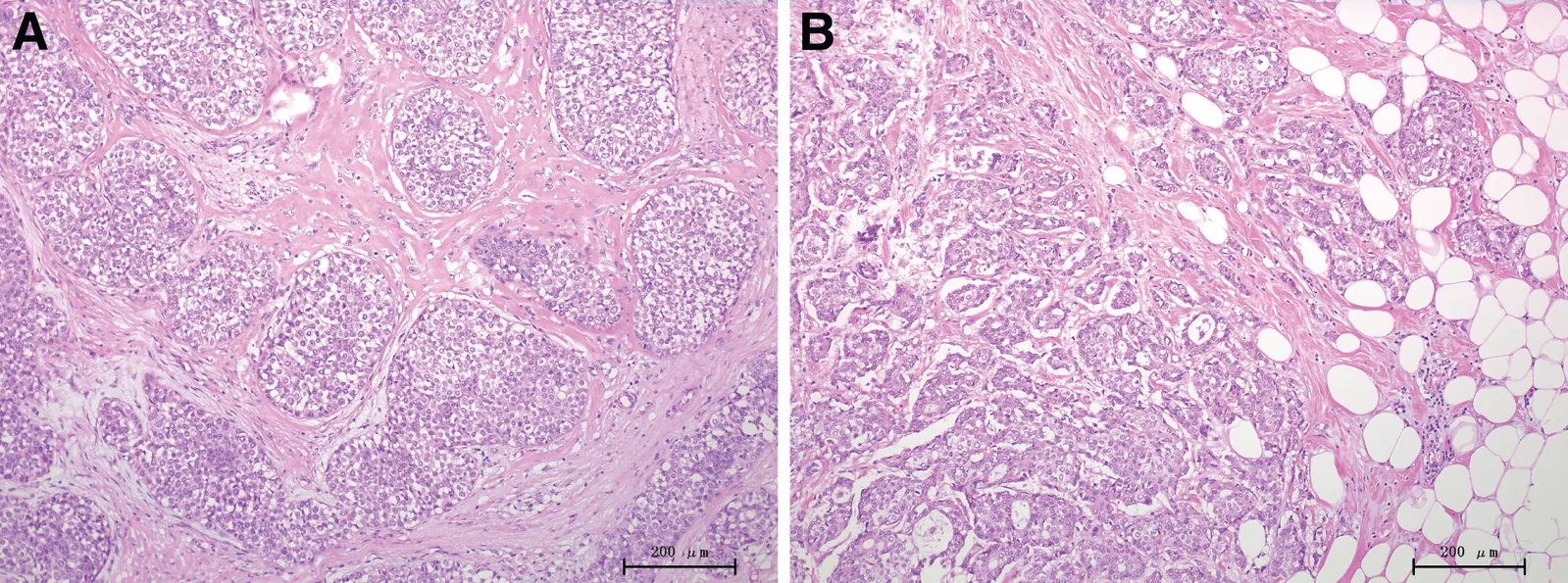
(**A**) Image of lobular carcinoma *in situ*. (**B**) Image of invasive ductal carcinoma.

When she was referred to our hospital for the new left-sided lesion, we used ultrasonography to visualize an ill-defined mass with low echogenicity, measuring 1.5 by 0.9 cm by 1.2 cm in the left upper-outer quadrant. Ultrasonography-guided core-needle biopsy specimens revealed ER-positive, PR-positive, and HER2-negative IDC. Genetic testing for *BRCA1* and *BRCA2* mutations was negative. Enhanced MRI of the breast and the head, together with FDG-PET–CT, showed no evidence of metastases, but preoperative SLN radioisotope mapping identified contralateral (right) axillary SLNs (**Fig. 2**). For this patient with a clinical stage IA, cT1cN0M0 BC, we decided to proceed with left mastectomy. Interestingly, the nodes did not stain during intraoperative SLN mapping using indigo carmine. Both the radioisotope and the indigo carmine were injected intradermally near the areola of the left nipple. Four SLNs were identified by 1-step nucleic acid amplification test, including 1 macroscopic metastasis and 2 microscopic metastases. One macroscopic metastasis showed a tumor load of 200000 copies/μL. Based on the contralateral location of the SLNs identified on lymphoscintigraphy, the presence of macroscopic metastasis in 1 SLN, and the high tumor load in the SLN with macroscopic metastasis, we proceeded with right ALND. We did not perform ipsilateral axillary surgery because the preoperative imaging showed no findings suggestive of lymph node metastasis on that side, and the repeat SLN biopsy revealed altered lymphatic pathways, clarifying that the ipsilateral axilla did not contain regional lymph nodes. The resected mastectomy specimen contained a 4-cm IDC lesion (**Fig. 1B**). Three lymph node metastases, all SLNs, were identified among 18 resected axillary lymph nodes. The Oncotype DX Breast Recurrence Score was 18. Because of the markedly different histology (LCIS vs IDC), initially negative surgical margins, the main locations of the primary and secondary tumors (central vs left upper-outer quadrant), the negative lymph node involvement of the primary tumor, and an extended disease-free interval (14 years) between the primary and secondary tumors, we diagnosed the secondary tumor as a new primary BC with regional lymph node metastases to the contralateral axilla, yielding a pathologic stage of IIB, pT2N1M0. Therefore, adjuvant therapy aimed at eradication was planned: chemotherapy with docetaxel plus cyclophosphamide, followed by endocrine therapy with tamoxifen and leuprorelin acetate, along with 1 year of tegafur/gimeracil/oteracil potassium. The patient had not experienced recurrence at the time of her most recent follow-up visit, 8 months after surgery.

**Fig. 2:**
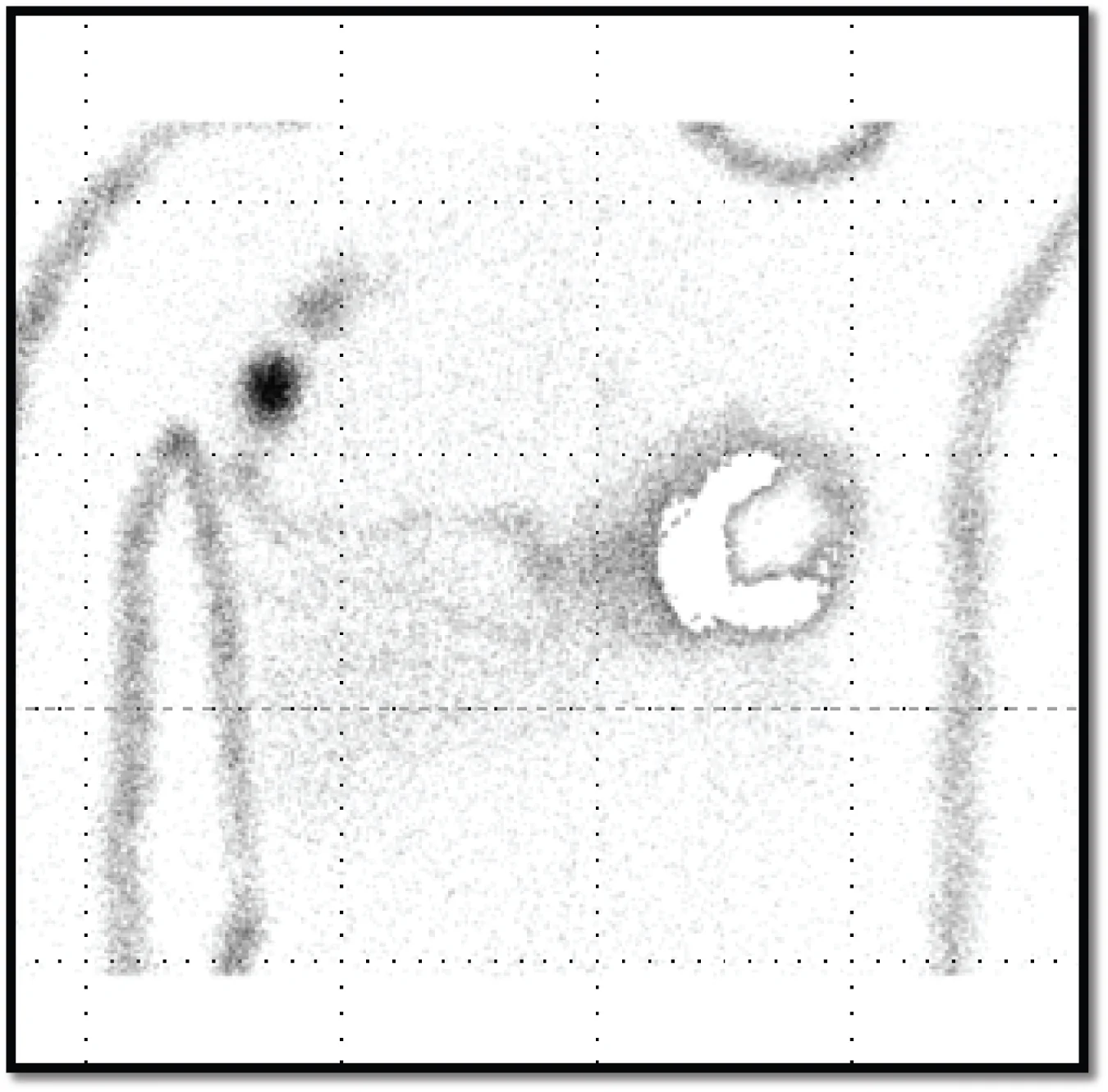
SLN radioisotope mapping identified contralateral (right) axillary SLNs. SLN, sentinel lymph node

## DISCUSSION

Because IBTR includes both true recurrences and new primary tumors, it is important to explain our rationale for diagnosing a new primary in our patient. The classification criteria used to distinguish these entities include the location of the primary and secondary tumors, status of the initial surgical margins, pathologic features such as lymph node positivity, and the disease-free interval.^4)^ New primaries are characterized by a lower rate of lymph node metastasis (8.7% vs 37.8%) and longer disease-free survival (mean, 62.1 months vs 46.6 months) compared with true recurrences.^4)^ In contrast, ER status was not useful for distinguishing between the entities in this patient, as both of her tumors were positive. Our patient had different anatomic locations of the main lesions, an extended disease-free interval, negative margins and negative lymph node metastasis with the primary tumor, and distinctly different morphology between the tumors. Therefore, the secondary tumor was judged to be a new primary.

It is necessary to discuss why we chose ALND instead of RT for our patient. In the AMAROS and OTOASOR clinical trials, which compared ALND against SLN biopsy plus RT for regional lymph nodes, both groups had low rates of regional lymph node recurrence, distant recurrence, and BC-related death, and there was no difference in overall survival.^5,6)^ It is also worth noting that ASCO guidelines recommend ALND if mastectomy was performed for cN0 with at least 3 positive SLNs, because patients with 3 positive nodes were underrepresented in trials evaluating the omission of completion ALND.^7)^ A separate report showed a non-SLN metastasis rate of 41.0% if the tumor load of the SLN metastasis was 15000 copies/μL or more, and a non-SLN metastasis rate of 17.4% if the SLN tumor load was less than 15000 copies/μL.^8)^ Given our patient’s SLN status, we did not think it was appropriate to omit contralateral ALND in favor of regional lymph node RT for our patient.

The clinical utility of repeat SLN biopsy is controversial because it is unclear whether contralateral lymph node metastases in patients with IBTR represent a regional event or distant metastatic disease. A systematic review of contralateral lymph node recurrence detected by repeat SLN biopsy in patients with IBTR showed an overall survival of 83.3% at a mean follow-up period of 27 months.^9)^ Because these numbers are not comparable to those seen with distant metastatic disease, we recommend planning a curative approach for regional lesions. However, our patient had noninvasive carcinoma during her first bout of BC and IDC for her current diagnosis; in her case, with different histologic patterns, the contralateral lymph node metastases should be thought of as being derived from a second, new primary BC. Yet if the contralateral lymph node pathways had developed as a result of her previous RT, these metastases would be considered a regional event. Repeat SLN biopsy would then play a key role in detecting regional lymph node position and status and in planning curative adjuvant treatment.

Our decision to omit postoperative RT to the right axillary region warrants discussion. Postmastectomy RT for patients with 1 to 3 positive nodes is recommended in domestic and overseas guidelines, such as those from NCCN, ESMO, and ASCO.^2,10–12)^ However, these guidelines are designed for patients with ipsilateral lymph node metastasis; there is no consensus on the use of RT for patients with contralateral regional lymph nodes. The ESMO guideline states that the resected part of the axilla should not be irradiated after ALND, except when there is clear residual disease after surgery.^11)^ We searched the Ichushi database, run by the nonprofit organization the Japan Medical Abstracts Society, using the terms: contralateral axilla, tumor metastases, or metastases, and excluding conference abstracts. Of the 10 Japanese case reports published between 2013 and 2025, only 2 described RT for contralateral regional lymph nodes (**Table 1**^13–22)^). We did not recommend RT for our patient with IBTR because there is no established evidence or views about the risk-vs-benefit of RT for contralateral regional lymph nodes. Since our patient had already received RT after BCS for her initial lesion, additional RT to the ipsilateral chest wall was not justified for her current BC.

**Table 1:** Characteristics of Japanese case reports

Age	Sex	1st BC age	Histology (1st BC)	SLN biopsy	ALND	RT	CxT	ER	PR	HER2	Histology (2nd BC)	Repeat SLN biopsy	Repeat SLN biopsy method	Contralateral lymph node metastases	Contralateral ALND	Contralateral RT	ER (2nd BC)	PR (2nd BC)	HER2 (2nd BC)	Reference
80’s	Female	60’s	Unknown	Yes	No	Yes	No	Positive	Positive	Negative	IDC (scir)	Yes	Dye + RI	No	No	No	Positive	Positive	Negative	^13)^
50’s	Female	18 years ago	IDC (solid)	No	Yes	Yes	Yes	Unknown	Unknown	Unknown	IDC (pap-tub)	No	Not done	Yes	Yes	No	Positive	Negative	Negative	^14)^
57	Female	46	IDC (solid)	No	Yes	Yes	No	Positive	Positive	Negative	Invasive micropapillary carcinoma	No	Not done	Yes	Yes	Yes	Positive	Positive	Negative	^15)^
58	Female	53	Paget’s disease	Yes	No	Yes	No	Negative	Negative	Positive	IDC (scir)	Yes	Dye + RI	Yes	Yes	No	Negative	Negative	Positive	^16)^
63	Female	52	DCIS	Yes	No	Yes	No	Negative	Negative	Not assessed	IDC with Paget’s disease	Yes	Dye + RI	Yes	No	Yes	Positive	Positive	Negative	^17)^
65	Female	58	IDC (scir)	No	Yes	No	Yes	Positive	Positive	Negative	IDC (solid)	No	Not done	Yes	Yes	No	Negative	Negative	Negative	^18)^
43	Female	33	IDC	No	Yes	Yes	Yes	Negative	Negative	Negative	IDC (scir)	Yes	RI	Yes	Yes	No	Positive	Positive	Negative	^19)^
51	Female	35	IDC (scir)	No	Yes	Yes	Yes	Positive	Positive	Negative	ILC	Yes	Dye	Yes	Yes	No	Positive	Negative	Negative	^20)^
71	Female	58	Unknown	No	Yes	Yes*	Yes*	Unknown	Unknown	Unknown	IDC	Yes	Dye + RI	Yes	Yes	No	Negative	Negative	Positive	^21)^
54	Female	48	IDC (pap-tub)	Yes	No	Yes	No	Positive	Positive	Negative	IDC (scir)	Yes	RI	Yes	Yes	No	Positive	Positive	Negative	^22)^

*Not completed by patient preference.

ALND, axillary lymph node dissection; BC, breast cancer; CxT, chemotherapy; DCIS, ductal carcinoma *in situ;* ER, estrogen receptor; HER2, human epidermal growth factor 2; IDC, invasive ductal carcinoma; ILC, invasive lobular carcinoma; pap-tub, papillotubular type; PR, progesterone receptor; RT, radiation therapy; RI, radioisotope; scir, scirrous type; solid, solid-tubular type; SLN, sentinel lymph node

When right axillary lymph node involvement is seen in a patient with a lesion of the left breast, surgeons must consider the possibility of a right-sided BC. In our patient, there was no evidence of a right-sided tumor on preoperative right breast mammography, ultrasonography, MRI, or FDG-PET–CT. Therefore, we determined that the most likely situation was contralateral axillary SLN metastases derived from a second left-sided primary BC.

## CONCLUSIONS

Repeat SLN biopsy may alter staging, directly influencing adjuvant treatment selection. Radioisotope mapping may be particularly important for patients with altered lymphatic drainage after surgery and RT. In this setting, contralateral axillary lymph node metastasis should not automatically be regarded as distant metastasis. Therefore, repeat SLN biopsy for IBTR should be considered.

## DECLARATIONS

### Availability of data and materials

The datasets supporting the conclusions of this article are included within the article.
